# Optimising human rabies vaccine supply chains: A modelling study

**DOI:** 10.1016/j.vaccine.2025.127108

**Published:** 2025-04-23

**Authors:** Martha M. Luka, Elaine A. Ferguson, Eleanor Rees, Husna Hoffu, Joel Changalucha, Kennedy Lushasi, Lwitiko Sikana, Mumbua Mutunga, SM Thumbi, Katie Hampson

**Affiliations:** a School of Biodiversity, One Health & Veterinary Medicine, College of Medical, Veterinary and Life Sciences, University of Glasgow, Glasgow G12 8QQ, Scotland, UK; b Environmental Health and Ecological Sciences Department, Ifakara Health Institute, Dar es Salaam, Tanzania; c College of Veterinary Medicine & Biomedical Science, Sokoine University of Agriculture, Morogoro 67804, Tanzania; d Centre for Epidemiological Modelling and Analysis, University of Nairobi, Nairobi, 00202, Kenya; e Institute of Immunology & Infection Research, School of Biological Sciences, University of Edinburgh, Edinburgh EH9 3FL, Scotland, UK; f Paul G Allen School for Global Health, Washington State University, Pullman, WA 99164, USA

**Keywords:** Rabies vaccines, Supply chains, Post-exposure prophylaxis, Zero by 30, NTDs

## Abstract

**Background:**

Rabies causes thousands of deaths annually in low- and middle-income countries. Despite effective vaccines for post-exposure prophylaxis (PEP), their expense, coupled with supply chain failures, leads to stockouts and preventable deaths. Investment by Gavi, the Vaccine Alliance, aims to improve access to post-exposure vaccines. We evaluate PEP demand in Tanzania and Kenya and examine stock management strategies for improving supply chains in Gavi-eligible countries.

**Methods:**

We fitted negative binomial distributions to five years of bite patient data from Tanzania (6646 patients, 20 districts) and Kenya (199,112 patients, 47 counties) to parameterise simulations of post-exposure vaccine demand under WHO-recommended intramuscular (IM) and intradermal (ID) regimens. We compared simulated vaccine use, stockouts, and the impact of stock management strategies across the observed range in demand.

**Results:**

Bite patient incidence varied dramatically; demand surges far exceeded monthly averages (in 6 % of months exceeding 3× average monthly bite patient presentations) and were most extreme in low-incidence settings. ID vaccination reduces vial use by >55 % and reduces stockout risk. Under ID vaccination vial savings are greatest in high-throughput settings, whilst risk mitigation is maximised in low-throughput settings. Decentralizing PEP to more facilities improves access, though reduces vial-sharing opportunities and so increases vial use. Resilient supply chain strategies were identified according to patient throughput, allowing for adaptation to changing demand.

**Conclusions:**

ID vaccination reduces vial use and stockouts, even in low-throughput settings. Tailoring stock management—through adjusted alert thresholds and restocking volumes—can simplify the integration of rabies vaccines into essential immunisation supply chains, improving their availability and preventing unnecessary deaths. However, logistical trade-offs must also be considered.

## 1 Introduction

Rabies remains a major public health challenge in many parts of the world, particularly in low- and middle-income countries (LMICs), where the disease continues to cause high mortality and morbidity [1,2]. Despite effective vaccines for humans and animals, rabies circulation persists due to limited implementation of dog vaccination [3], and human deaths continue due to poor access to post-exposure prophylaxis (PEP) [4]. PEP consists of wound washing, timely administration of a course of post-exposure vaccines, and, where indicated, Rabies Immunoglobulins (RIG) [5]. For high-risk bite victims who do not receive PEP, one in five will likely develop rabies, a disease that is nearly universally fatal [6]. Although an emergency medicine, rabies postexposure vaccines are not well managed, and supply chain inefficiencies have catastrophic implications [6–8]. Bite patients often face high out-of-pocket costs and travel long distances in search of these vaccines, which present access barriers, especially for those in rural and impoverished communities [7,9,10]. For instance, PEP costs in Tanzania exceed USD 70 per bite victim [2], and in Kenya, obtaining a single vaccine dose costs between USD 8 and 120 [11].

In response, the ‘Zero by 30’ global strategic plan seeks to eliminate dog-mediated human rabies deaths by 2030 [12], recognising the crucial role of PEP and mass dog vaccination [1,7]. PEP remains the only method guaranteed to prevent the onset of clinical symptoms following exposure to rabies [5], and missed post-exposure vaccination is the cause of almost all human rabies deaths [6,13,14]. Despite this, PEP access remains limited [10], with vaccines often placed only in urban centres and supply chains not responsive to demand. Financial barriers and poor monitoring further contribute to lengthy stockouts and the limited availability of PEP outside of major cities [3,4,15].

To address these issues, Gavi, the Vaccine Alliance, incorporated human rabies vaccines in its 2021 Vaccine Investment Strategy [16–18]. This signified a strategic investment that aims to enhance healthcare-seeking behaviour and improve access by making post-exposure vaccines free of charge to bite patients. Gavi advocates for dose-sparing intradermal (ID) administration of rabies vaccines [5,19], which allows multiple patients to be vaccinated from a single vial 18. The World Health Organization (WHO) recommends an abridged ID regimen, IPC (Institut Pasteur du Cambodge), which requires only 0.2 ml per dose, much less than the commonly used intramuscular (IM) regimens, where a single dose consumes an entire vial, typically 1 ml in size [5]. ID vaccination can be completed in 1 week, requiring a total of three visits, making it a less expensive and logistically preferable option compared to alternatives. Due to contamination concerns, opened vials must be discarded at the end of each day, which may limit vial-sharing opportunities under ID vaccination in low-demand settings. This raises questions about the benefits of ID vaccination if vial sharing opportunities are limited.

The management of rabies post-exposure vaccines in most countries is outside national vaccine supply chains, such as the Expanded Programme on Immunisation (EPI), with Bhutan being a notable exception [10]. This separation poses logistical challenges, and limited cold chain capacity is cited as a barrier to storing PEP alongside childhood vaccines [6]. WHO recommends integrating PEP into routine immunisation services to benefit from strengthened supply chains and, therefore, improve distribution and availability [20,21]. A centralized procurement and logistics management system also assures the quality of vaccines and gives a better negotiating power for the bulk purchase of vaccines [22]. Unlike routine vaccines, with predictable, demographic-driven demand [23], rabies PEP demand varies with disease dynamics and healthcare-seeking behaviours [6]. Moreover, rabies PEP is required as an emergency medicine that needs to be administered without delay. These characteristics mean that there is a need for tailored operational guidance to manage rabies PEP supply. Gavi’s support for EPI integration seeks to address these challenges, but its operationalisation requires overcoming obstacles related to training, intersectoral coordination, and mitigating potentially higher wastage risks associated with ID vaccination. Gavi’s learning agenda, reinitiated in 2023, emphasises identifying strategies for effectively integrating rabies PEP into EPI while ensuring the program’s success.

In addition, uncertainties remain about how Gavi’s investment will impact healthcare-seeking patterns in these endemic settings. This highlights the need for resilient supply chains that can efficiently meet changing demand and ensure consistent availability of these emergency vaccines [7]. Our research addresses the unique challenges of PEP demand by evaluating middle and last-mile supply chain strategies, examining how restocking approaches can meet this demand and exploring options for integration of PEP within national immunisation supply chains. We provide insights into stock management at sub-national levels, where issues like decentralization and vial-sharing influence vaccine availability and wastage.

## 2 Methods

### 2.1 Data sources

We used data from an Integrated Bite Case Management (IBCM) platform in Tanzania [24]. These data, collected between June 2018 and December 2023, were the foundation for developing our models. We also compared PEP demand patterns in Tanzania with Kenya to assess the applicability of PEP policies across the broader region. In both countries, human rabies vaccines are classified as non-routine and are not anchored within national immunisation programs. Procurement is typically reactive, driven by local outbreaks, and based on historical consumption data. As a result, stockouts are frequent and can be prolonged. Both countries, with endemic rabies, diverse populations, and geographies, are Gavi-eligible and could benefit from investment to improve rabies vaccine access.

IBCM is a surveillance approach that links public health and veterinary workers to assess rabies risk in animal bite patients and investigate biting animals [24–26]. From mid-2018, IBCM was introduced to 20 districts in Tanzania (average district population 320,000, ranging from 65,000 to >1.6 million) across four regions: Mtwara, Mara, Lindi, and Morogoro. These regions had a combined population of 7,100,000 in 2019, and an estimated human-to-dog ratio (HDR) of 30:1 [24,27]. Bite patient data from the IBCM platform in Tanzania included patient demographic details, risk assessment status, and PEP provided. IBCM was established at government public health facilities designated to stock PEP and therefore excludes bite victims who sought care from facilities that do not routinely stock PEP or from private healthcare facilities [24,25].

In Kenya, data were from the Kenya Health Information System (KHIS), a national repository for health services. The KHIS dataset includes facilities that stock PEP, such as county and sub-county hospitals; as well as private hospitals; and lower-level facilities, like rural health clinics, that do not typically stock PEP. This dataset, collected between January 2021 and March 2024, aggregated at the sub-county level (*n* = 324, average population 180,000, ranging from <500 to >1 million), includes dog bite cases regardless of whether patients received PEP.

### 2.2 Post-exposure vaccine demand

We analysed all recorded bite patient data to characterise spatiotemporal variation in PEP demand. We compared trends over time and mapped the frequency of patient presentations across administrative levels. To quantify monthly variability in bite patient presentations, we calculated a ‘surge factor’, defined as the ratio of the 97.5 % quantile of monthly bite cases to the mean in each district. A locally estimated smoothing method (LOESS) was applied to visualise trends and examine the relationship between surge factors and mean monthly bite cases. We fitted a negative binomial distribution to each district’s monthly bite presentations, capturing the overdispersion in bite counts.

The fitted (mean and dispersion) parameters were then used to simulate a time series of monthly bite patients for each district. For IBCM districts, simulations were based on district-specific mean and dispersion parameters estimated from the IBCM data. For hypothetical settings, we explored a range of means, drawing the dispersion parameter at random from a uniform distribution within the range observed from IBCM data. We developed an algorithm to compare vial use under two WHO-recommended PEP regimens [5]: the IM 4-dose regimen (used in most Gavi-eligible countries) and the abridged 1-week ID regimen, which is proposed for use with Gavi-supported vaccines (Supplementary Table 1) [19].

Our algorithm involved (1) simulating monthly bite patients by district using negative binomial distributions; (2) assigning patient presentation dates within months using a uniform random distribution; (3) generating patient return dates based on specified regimens (Supplementary Table 1); (4) calculating daily vial use, accounting for discard of incompletely used vials at the end of each day; and (5) iterating (steps 1–4) 1000 times to capture variation.

Given the high-risk context of rabies-endemic areas and limited diagnostic confirmation, we assumed that all bite patients receive an entire course of PEP regardless of whether the bite was from a suspected rabid animal or not. Rabies Immunoglobulin (RIG) was not included in this analysis as Gavi’s investment focuses on post-exposure vaccines, and RIG is rarely available in these endemic countries. We also assumed that post-exposure vaccines were available and that for ID vaccination, each 1 ml vial could administer up to five 0.2 ml doses.

### 2.3 Decentralizing access

We analysed the impact of decentralizing PEP on vial use across three scenarios. These scenarios were informed by current practices in Tanzania, where post-exposure vaccines are typically centralized in district hospitals, but demand pressure led to more decentralized provisioning in some districts, though with availability constrained by local government funds (Supplementary Table 2). The scenarios evaluated were:

a) No decentralization: Vaccines are exclusively available from a single health facility. This centralized scenario maximises vial sharing but may increase travel time and indirect costs for patients, affecting timely vaccine access.
b) Moderate decentralization: Vaccines are accessible at a central hospital and three satellite health facilities. We assume that most (70 %) bite patients seek care at the central hospital, while 30 % attend satellite clinics. This scenario expands access to more points of care, potentially reducing patient travel time and costs.
c) High decentralization: Vaccines are modelled to be available at a central hospital and seven satellite health facilities. We assume 50 % of bite patients seek healthcare at the central hospital, while the remainder attend the satellite clinics, aiming to maximise accessibility and minimise patient travel and costs. Although only a few districts have this level of decentralization, this scenario is intended to model the impact of further expanding PEP access.

To further explore how decentralization may influence demand, we modelled scenarios where patient throughput increased by 10 % and 25 % across both low- and high-throughput districts. This sensitivity analysis aimed to reflect potential increases in care-seeking behaviour that may result from improved geographic access and to estimate the corresponding changes in vial needs.

### 2.4 Supply chain management

To investigate approaches to supply chain management, including integration within supply chains for childhood vaccines, we explored resupply criteria, specifically analysing i) an alert threshold below which restocking is triggered and ii) the restocking volume. We searched for suitable values for these criteria to accommodate varying patient throughput across settings, enabling responsive vaccine supply to demand fluctuations.

**Fig. 1:**
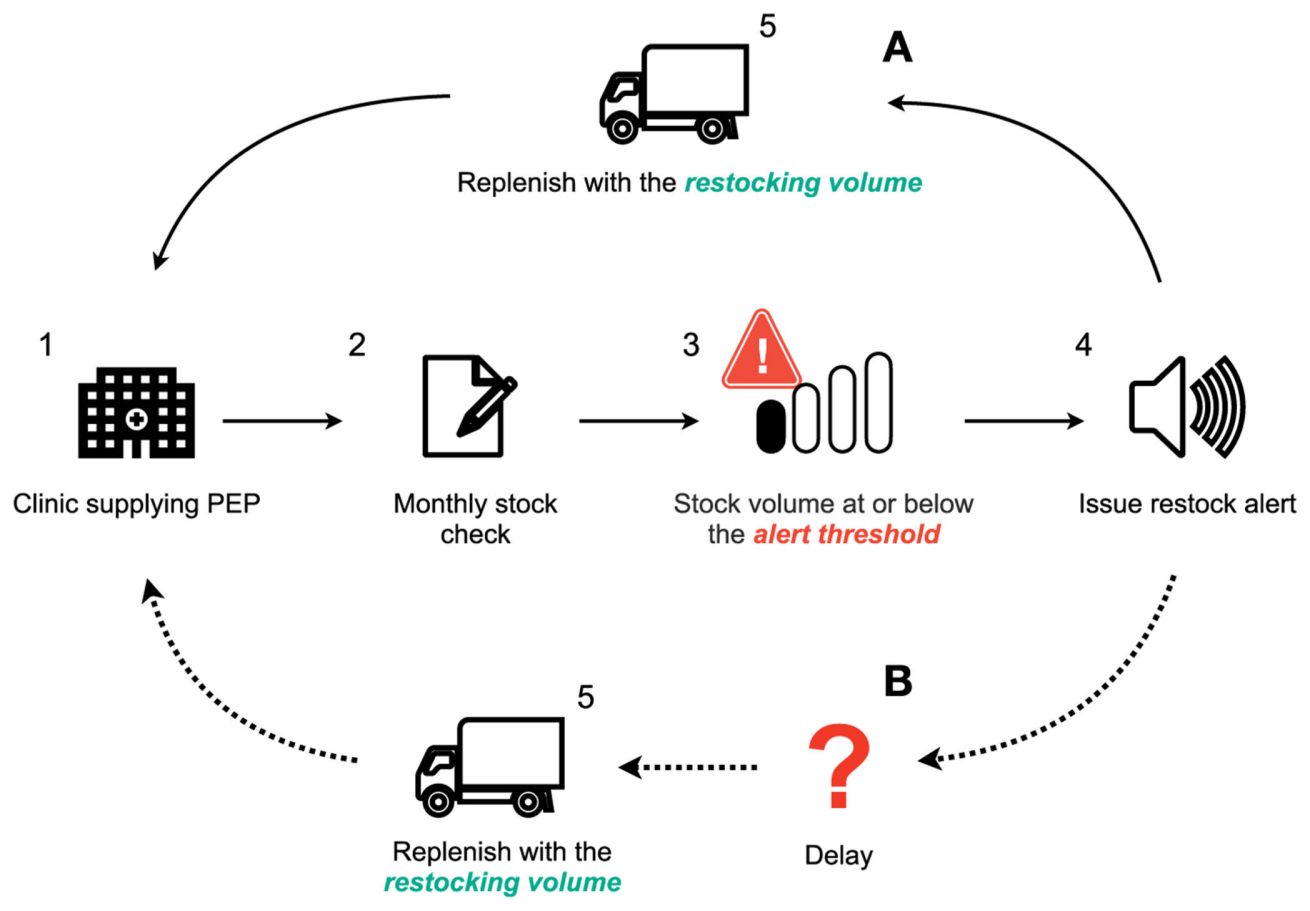
Scenarios for rabies post-exposure prophylaxis (PEP) vaccine supply chain and stock management. Following a stock alert, restocking can occur via two scenarios: (A) prompt restocking or (B) delayed restocking after a lag.

We explored the impact of restocking delays on vaccine availability and the resilience of the supply chain to meet demand under i) ideal stock management systems and ii) constraints that may reflect more realistic vaccine supply chain operations (Fig. 1). Specifically, we compared prompt restocking following an alert (i.e., if an alert is raised in month *t*, restocking occurs in month *t* + 1), versus delayed restocking with varying lags after an alert (e.g., restocking occurs in month *t* + 2 for a 1-month delay or *t* + 3 for a 2-month delay). In Tanzania, the Immunisation and Vaccine Development (IVD) program requires monthly stock reporting for routine vaccines and typically restocks quarterly, with provision for additional deliveries in response to stockouts.

Restocking volumes were set to exceed the alert threshold by at least four times the district’s average monthly bite cases. This multiplier, derived from vials required per patient under the IM regimen, was consistently applied across all regimens for simplicity. The lowest feasible restocking volume and corresponding alert threshold that ensured that no stockouts occurred in less than 5 % of simulations were selected.

Using these optimised thresholds and volumes (e.g., 75 vials with a 50-vial threshold for low-throughput settings and 200 vials with a 110-vial threshold for high-throughput settings), we simulated stockouts under a range of restocking delays. Scenarios were stratified by setting: low-throughput districts (mean < 5 monthly bite patients) and high-throughput districts (mean 10-20 monthly bite patients). The proportion of simulations that resulted in stockouts was calculated under each scenario to evaluate how restocking delays impact vaccine availability and supply chain resilience.

## 3 Results

### 3.1 Post-exposure vaccine demand

From June 2018 to December 2023, IBCM data were collected on 6973 bite patients across 20 districts in Tanzania. After excluding dispensaries (*n* = 8; 229 patients) and clinics that temporarily stocked PEP (*n* = 13; 121 patients), we analysed data from 6608 patients presenting at 55 health facilities. The mean annual incidence of bite patient presentations was 20.38 per 100,000 population (range 2.21–102.95), with marked geographical variation across districts. Mean monthly bite presentations also varied considerably, ranging from 0.34 to 20.76 patients per district, Supplementary Table 2. Of the 20 districts, 14 (70 %) were classified as low throughput (<5 mean monthly bite presentations), 4 (20 %) as medium throughput (5–10 mean monthly bites), and 2 (10 %) as high throughput (>10 mean monthly bite presentations). For example, Liwale, a low-throughput district, averaged 1.03 bite presentations per month (range 0–7), whereas Ulanga, a high-throughput district, averaged 15.39 presentations per month (range 1–49).

**Fig. 2:**
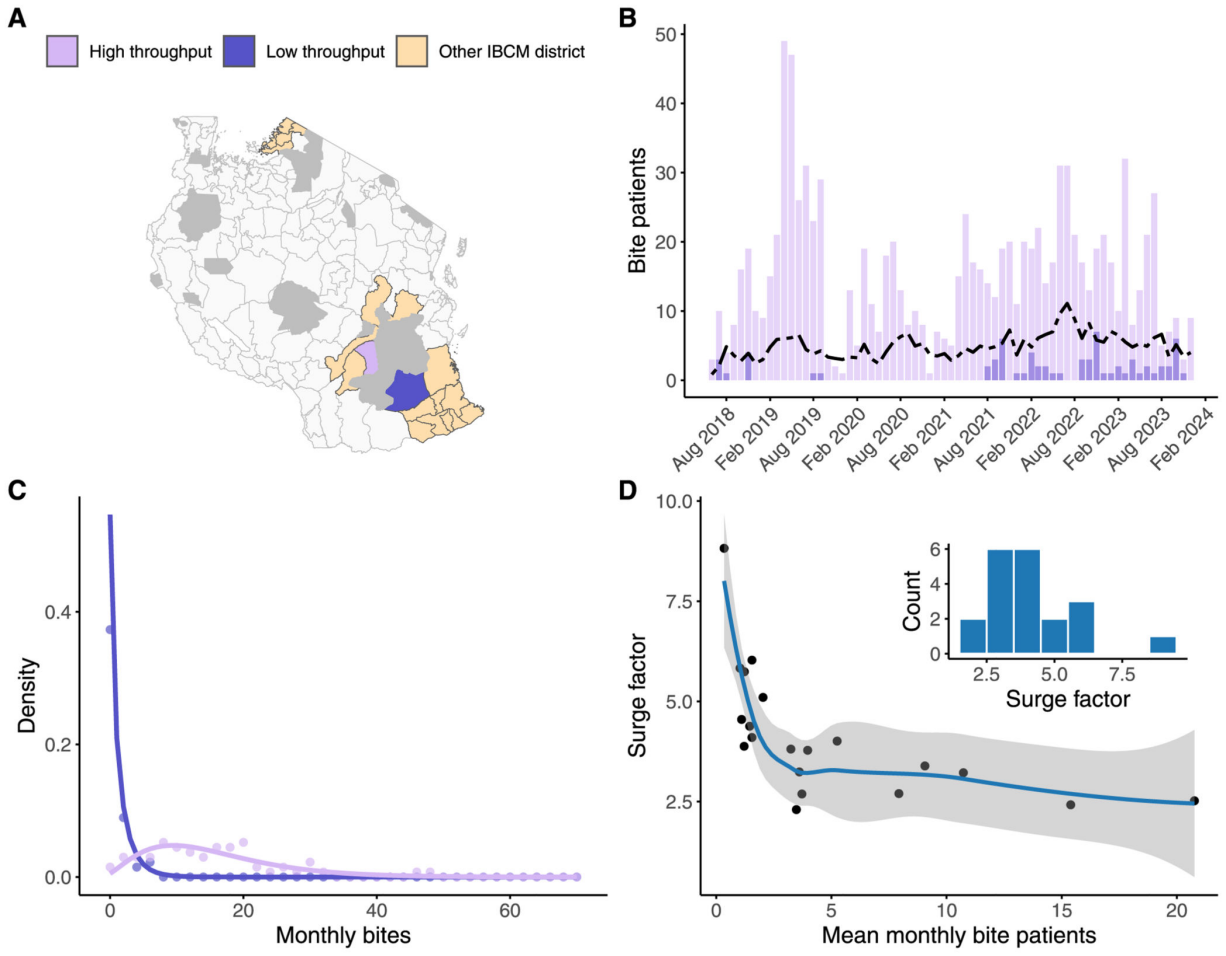
Bite patient dynamics across IBCM districts in Tanzania. (A) IBCM districts (yellow), showing examples of a high-throughput district (Ulanga) and a low-throughput district (Liwale). Grey areas indicate wildlife protected areas. (B) Time series of monthly bite patient presentations, comparing the example high-throughput and low-throughput districts. The dotted line represents the average monthly bite patient count across all IBCM districts. (C) Distribution of monthly bite patients (points) with a fitted negative binomial distribution (solid lines) for both the low-throughput and high-throughput districts. (D) Relationship between mean monthly bite patients and the demand surge factor with the line indicating the trend, and the shaded polygon representing the 95 % confidence interval around this trend; the inset shows the distribution of surge factors across districts. (For interpretation of the references to colour in this figure legend, the reader is referred to the web version of this article.)

Temporal variation in bite patient numbers was notable. Districts such as Liwale and Malinyi reported no bites for more than 50 % of the months. Yet, rabies-related deaths did not necessarily correlate with lower bite reports (zero in Liwale, seven in Malinyi). Some districts, such as Newala, experienced surges up to ten times their monthly average (Supplementary Table 2; Supplementary Fig. 1). While there were notable surges in specific months — for example, February 2023 in Bunda and June 2022 in Nanyumbu — we did not observe any consistent seasonal patterns in patient presentations across districts (Supplementary Fig. 1). Overall, bite patient presentations followed a negative binomial distribution, with a high frequency of zero-bite months in low-throughput districts, Fig. 2.

Data from Kenya revealed similar variation, covering 199,112 bite patients across 47 counties and 324 sub-counties. The average annual incidence of bite patients was 140.52 per 100,000, with substantial variation between sub-counties (5.28 to 384.14 per 100,000). Surge factors also ranged from 1.4 to 19.5 (mean 2.59), and low and high-throughput sub-counties followed similar negative binomial bite distributions to those fitted for Tanzanian districts, Supplementary Figs. 2 and 3.

As the average monthly bite presentations increased in both countries, the surge factor—indicating sudden increases in bite patients—tended to decrease. This trend was most pronounced in locations with fewer than five bite patients per month, where surge factors were highest. In locations with higher throughput (>8 bites per month), the surge factor stabilised between two and four.

**Fig. 3:**
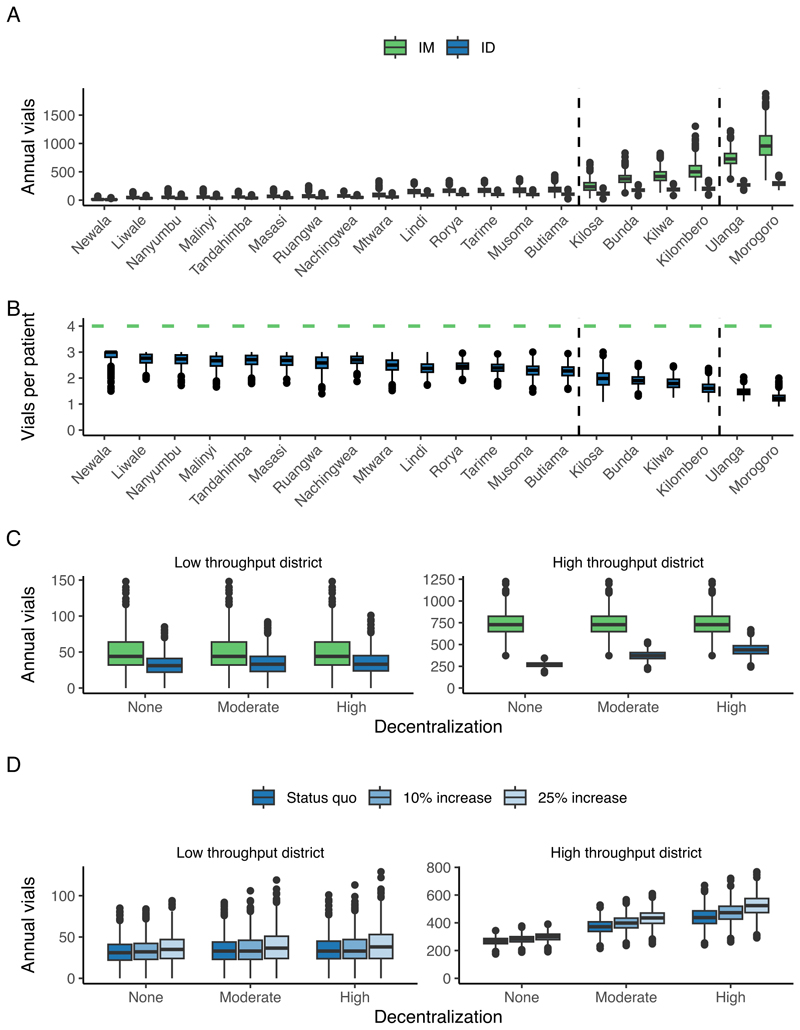
Predicted annual vial requirements for IBCM districts in Tanzania. (A) Total annual vials required for IM and ID vaccination, assuming a centralized scenario. Each box plot represents the distribution of vial use across 1000 simulations, with 1 PEP facility per district. (B) Vials required per patient for IM and ID vaccination across the same districts and scenarios. Dashed lines separate low, medium, and high-throughput districts. (C) Annual vial requirements for low (Liwale) and high (Ulanga) throughput districts under decentralization scenarios (None, Moderate, High) and route of vaccination (IM and ID). (D) Annual vial requirements for ID vaccination under the same decentralization scenarios shown in (C), comparing vial use under the status quo and with 10 % and 25 % increases in patient presentations.

**Fig. 4:**
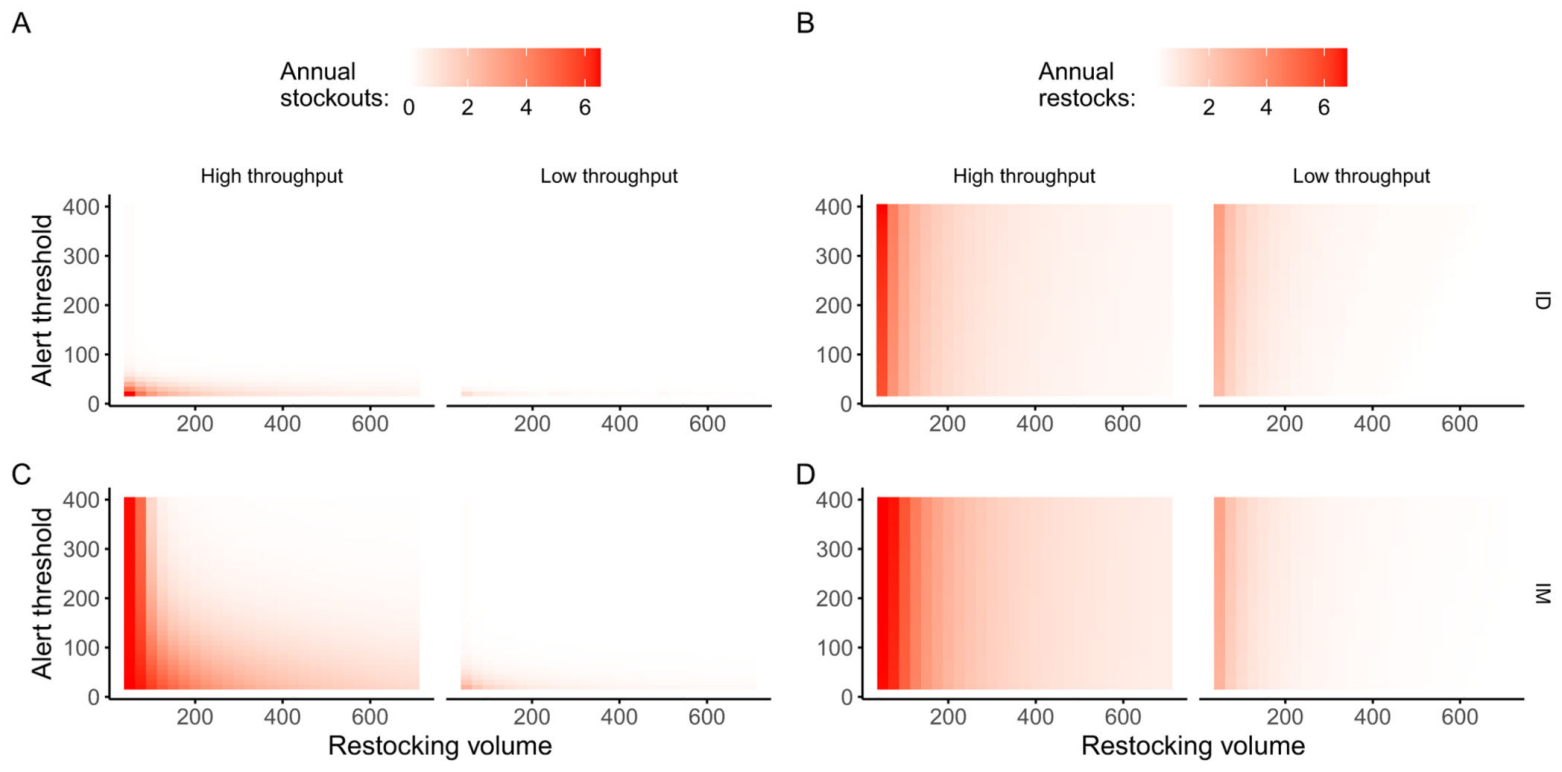
Predicted PEP stockouts and restocks under different supply chain criteria. Panels show the annual number of stockouts (A, C) and restocks (B, D) for high throughout (mean monthly bites = 20) and low throughput (mean monthly bites = 5) districts for ID (A, B) and IM (C, D) vaccination, under a centralized scenario with a 1-month restocking lag. Darker colours represent higher numbers of stockouts or restocks.

Simulations of vaccine demand under the centralized PEP scenario showed that the ID regimen notably reduced annual vial requirements compared to the IM regimen, particularly in high-throughput settings. In Ulanga, for instance, the IM regimen required an average of 739 vials per year (95 % C⋅I 372–1224), compared with 269 vials (95 % C⋅I 175–344) for the ID regimen, Fig. 3A. Across all IBCM districts, annual vial consumption was estimated at 2103 (95 % C⋅I 1252–3040) for the ID regimen, compared with 4705 (95 % C⋅I 2452–7625) for the IM regimen, representing a 55.3 % reduction. In low-throughput settings, opportunities for vial sharing were limited, resulting in minimal differences in vial usage between regimens. For these low-throughput settings under the centralized scenario, each patient required approximately three vials for the ID regimen versus four with the IM regimen. However, vial sharing was more frequent in high-throughput settings, reducing vial use to one per patient on average under ID vaccination (Fig. 3B) but not reaching the most efficient scenario of minimal vial use (0.6 vials per patient). The ID regimen provided a buffer during surges, allowing for multiple patients to share vials on the same day, Fig. 4.

### 3.2 Decentralizing access

During the study period, a mean of 2.75 facilities per district (range 1–7) provided post-exposure vaccines, reflecting moderate decentralization of services. Districts such as Liwale, Malinyi, and Ruangwa, with only one PEP-providing facility, aligned with the centralized model, where all patients depended on a single facility, whereas Morogoro, with seven facilities, represented a highly decentralized model. On average, 75.6 % of bite patients accessed PEP at the main district facility, although this proportion varied across districts (40–100 %). The number of facilities consistently offering PEP increased in nine districts because of government decentralization efforts. This expansion corresponded with a reduced proportion of patients visiting the main district facility in seven districts, Supplementary Table 2.

In simulations, decentralization had minimal impact on vial use in low-throughput settings due to the already low opportunities for vial sharing. In contrast, high-throughput settings, such as cities and regions with higher bite patient incidence, had more vial-sharing opportunities, which were reduced by increasing decentralization. However, even with high decentralization, simulated vial demand under ID vaccination remained lower than under IM, Supplementary Fig. 4. This is evident in high-throughput districts like Ulanga, where approximately 269 vials are required annually under a centralized scenario administering ID vaccination, with demand rising to 373 (95 % C⋅I: 273–474) vials with moderate decentralization and 440 (95 % C⋅I: 308–581) vials with high decentralization, Fig. 3C. Under the IM regimen, an average of 739 vials (95 % C⋅I: 372–1224) would be required annually, with decentralization having no impact on overall vial usage.

Increases in patient throughput under decentralization led to proportionally smaller increases in vial use. A 10 % rise in patients resulted in just a 3.1 % increase in vial use in low-throughput and 6.7 % in highthroughput districts, while a 25 % increase in patients led to 12.5 % and 17.2 % increases in vial use, respectively (Fig. 3D).

### 3.3 Supply chain management

We identified restocking volumes and alert thresholds that minimise stockouts across districts with varying patient throughputs. Under monthly restocking with a 1-month lag, districts with fewer than five bite patients per month required a minimum of 75 vials and an alert threshold of 50 vials to prevent stockouts. In contrast, IM vaccination in the same setting required 210 vials and a 160-vial alert threshold—nearly three times more than ID vaccination. For districts with 10–20 monthly bite patients, ID vaccination required 200 vials and an alert threshold of 110, while IM vaccination required 690 vials and a 580 vial threshold (Fig. 4).

We also provide quarterly restocking recommendations that align with quarterly restocking such as Tanzania’s IVD program. In Tanzania, maintaining supply with a < 5 % probability of stockout using ID vaccination requires a minimum restocking volume of 100 vials for low-throughput districts and 225 vials for high-throughput districts, with alert thresholds of 60 and 120 vials, respectively (Table 1).

**Fig. 5:**
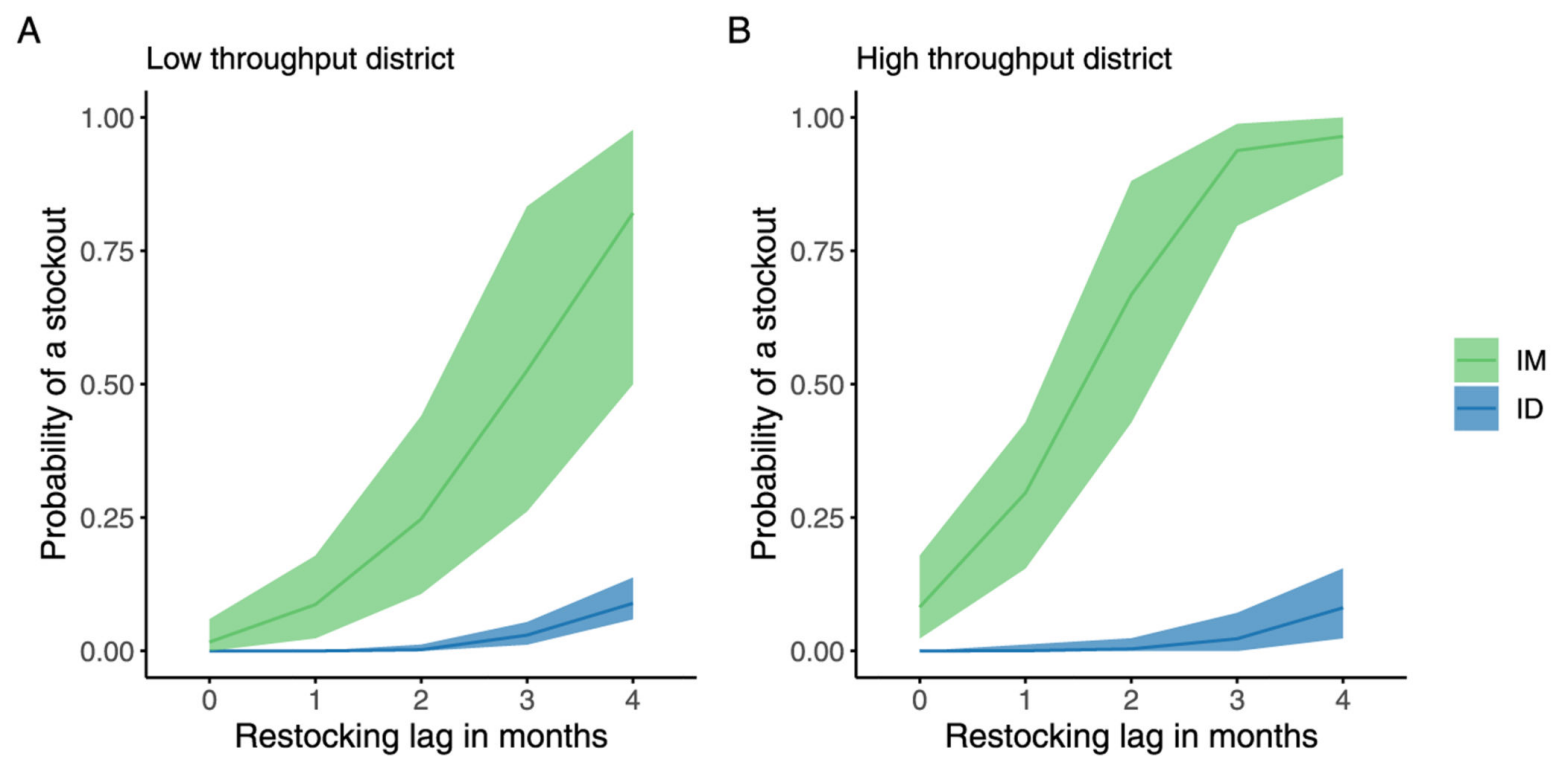
Relationship between restocking lag and probability of stockouts in A) low throughput and B) high throughput setting. Low-throughput settings are defined as those with fewer than 5 monthly bite cases on average, while high-throughput setting experience up to 20 monthly bite cases. The shaded areas represent the 95 % confidence intervals for the probability of stockouts under each scenario. Simulations were conducted using restocking volumes and alert thresholds optimised to keep stockout probabilities below 5 % with a 1-month restocking lag: 75 vials with a 50-vial alert threshold in a low-throughput setting and 200 vials with a 110-vial alert threshold in high-throughput settings.

Our simulations showed that stockouts increased with longer restocking delays in both high- and low-throughput settings, with ID vaccination demonstrating a greater resilience to stockouts compared to IM vaccination. By the fourth month of delayed restocking, the probability of stockouts exceeds 75 % for IM while remaining below 25 % for ID vaccination. In high-throughput settings, the stockout probability for IM vaccination increases sharply, exceeding 90 % by the third month of the restocking delay, whereas the stockout risk remains substantially lower for ID vaccination over the same period, Fig. 5.

## 4 Discussion

### 4.1 Key findings

Rabies continues to pose a critical public health challenge in many LMICs and limited access to PEP is a major cause of these preventable deaths. We report substantial spatial and temporal variability in bite patient incidence across East Africa, including surges exceeding ten times the monthly average—illustrative of unpredictable, localized rabies outbreaks. Vaccine supply chains need to be robust to these dynamics, including the required multi-visit regimens and the emergency nature of PEP demand.

We address these challenges by exploring supply chain strategies to maintain reliable PEP using extensive district-level data from Tanzania, complemented by comparative insights from Kenya. We show that adopting ID administration could optimise vaccine use and improve resilience to demand surges by buffering against stockouts, especially in low-throughput settings. Contrary to perceptions that ID vaccination is unsuitable for low-throughput settings due to limited vial-sharing opportunities, we show that the WHO-recommended 1-week abridged ID regimen reduces overall vial use by 25 % compared to IM, even with minimal vial-sharing.

We identify strategies for optimising stock management of these vaccines according to setting-specific throughput. For instance, we show that low-throughput districts in Tanzania (such as Liwale and Nanyumbu) require minimum restocking volumes of 100 vials and alert thresholds of 60 vials to trigger restocking assuming quarterly restocking, whereas higher-throughput districts like Ulanga need restocking with at least 225 vials according to an alert threshold of 120 vials for maintaining PEP availability. Stockouts due to unexpected surges in bite patient numbers may still occur; however, these can be avoided by setting an appropriate alert threshold that triggers ad hoc restocking outside the regular quarterly schedule.

Logistical and financial constraints often limit timely access to PEP, especially in rural and resource-constrained areas. Decentralizing health services can reduce travel burdens and enhance patient compliance with multi-dose regimens, as seen for rabies PEP [6,10]. This study showed reduced reliance on main district facilities after PEP centres expanded in seven of nine districts, demonstrating decentralization’s potential to enhance access. Even under anticipated increases in healthcare seeking with decentralized PEP access we did not predict very large changes in vial use because of corresponding increases in vial sharing. However, decentralization places additional demands on resources, for example, in maintaining the cold chain and training healthcare workers in ID administration. If the supply chain is not well managed, patients may still face stockouts and need to undertake extended travel to secure PEP, as shown by Tanzania’s IBCM data. The extent to which PEP should be decentralized to outlying facilities will depend on logistical constraints and requires a reliable supply. Gavi’s investment could address this challenge by aligning resources with decentralization goals to ensure equitable access [19]. A balanced strategy, selecting PEP distribution points for both accessibility and logistical feasibility, is needed.

### 4.2 Broader context

Our findings have broader implications beyond rabies PEP. The modelling algorithms developed in this study can be adapted to support the management of other emergency-use biologics with unpredictable demand, such as snake antivenoms [29], Ebola vaccines [30], and yellow fever vaccines—the latter of which has employed fractional dosing during outbreak responses [31]. These products face similar challenges with stockouts that can be life-threatening. Comparable issues also arise in livestock vaccines, such as for foot and mouth disease [32,33].

Dog bite patient presentations to healthcare facilities vary widely around the world, influenced by factors such as accessibility, costs, and community awareness [6,26,34]. For example, bite incidence rates of over 600/100,000 persons per year have been reported in the Philippines [34,35], compared to 20.48/100,000 persons/ year in Tanzania. Kenyan KHIS data showed higher bite patient numbers than Tanzanian IBCM data, likely due to inclusion of patients bitten by healthy animals, double-counting at facilities without PEP, and broader nationwide coverage, including urban areas with higher care-seeking rates. Similarly, higher numbers of bite patients are recorded in Tanzania’s electronic Integrated Disease Surveillance and Reporting (eIDSR) data, covering all healthcare facilities, including those not stocking PEP. While the KHIS data may be less precise in capturing rabies risk, it represents an upper bound of PEP demand, as it reflects all reported bite cases, including many who do not receive PEP and are unlikely to need it. Systems incorporating risk assessments, such as IBCM, may help to ensure judicious PEP administration by prioritizing high-risk exposures.

The considerable variation in bite patient incidence across our study settings also points to the need to understand how mass dog vaccination could impact PEP demand in the region. In Southeast Tanzania, demand increased across many districts after free dog vaccination was discontinued at the end of a donor-funded project, and a resurgence of rabies was seen [28]. Elsewhere in Tanzania, demand surges occurred during rabies outbreaks and have subsided following the local elimination of rabies through dog vaccination [25]. More generally, given that PEP demand will persist unless rabies is controlled at its source, sustained One Health strategies that scale up dog vaccination and human PEP access are crucial. Close monitoring of PEP use during Gavi’s rollout will be essential for refining demand forecasts, ensuring efficient vaccine management, and informing future exit strategies.

The relationship between PEP demand and dog vaccination, particularly as PEP becomes more accessible and supported by Gavi investment, remains unknown. Hence, there is an important need for close monitoring of PEP use during the Gavi rollout to improve future demand forecasts and vaccine management.

### 4.3 Strengths and limitations

A strength of our study lies in the use of spatially resolved data over multiple years, allowing us to capture the geographic and temporal variability of bite incidence and provide tailored recommendations. However, our analysis has limitations. Frequent stockouts in many facilities (only 48 % of IBCM facilities have never recorded a stockout) in Tanzania may have led to conservative estimates of PEP demand. Patients who face vaccine shortages, high costs, or accessibility challenges may forego PEP, leading to underreporting. Similar challenges have been observed in Kenyan facilities [15]. More broadly, the discrepancies between bite incidence data reported through the IBCM and KHIS systems highlight the need for effective monitoring of PEP use to meet unmet demand and improve forecasting. We focused on middle- and last-mile supply chains, assuming Gavi’s investment would address national-level availability. However, this assumption may warrant future review, as PEP demand is more volatile than that for routine vaccines.

For simplicity, we modelled 100 % patient compliance with multidose PEP regimens. However, future studies should also consider more realistic levels of patient compliance, which could influence demand and vaccine usage. We also did not examine how patient presentations vary on weekdays rather than weekends, which has been observed in some settings. Given the emergency nature of rabies PEP, clinics designated for PEP should operate seven days a week to ensure timely access. This is an important consideration for integrating PEP into essential immunisation services, given that these are typically offered in health facilities that open during restricted hours. While our study primarily focuses on how PEP demand can be met, operational aspects of integration with EPI require further investigation. Expanding data collection and modelling efforts to include these factors could further refine stock management strategies and support policy decisions.

### 4.4 Conclusions and recommendations

Gavi’s Vaccine Investment Strategy provides a timely opportunity to integrate rabies PEP into national immunisation programs. By adapting stock management to local demand and leveraging the dose-sparing benefits of ID vaccination, countries can enhance the efficiency and reliability of PEP delivery, supporting WHO’s recommendations of ID administration [5] as a practical and cost-effective solution. The demand dynamics that we report also highlight the challenges of managing rabies PEP as an emergency medicine, requiring stock management strategies fundamentally different from the more stable demands of routine immunisation programs. The variability in bite incidence and the potential for localized surges reinforce the need for dynamic stock management practices, particularly as PEP demand is expected to increase following Gavi’s strategic investment.

Integrating rabies vaccines into routine supply chains involves balancing optimal placement with baseline supply requirements. The volume of rabies vaccines needed at the district level is relatively small compared to other vaccines, potentially simplifying the integration process. However, practical experience of logistics challenges and resource implications should be considered. Finally, to enhance vaccine delivery and mitigate stockout risks, dynamic adjustments to middle-mile and last-mile logistics are essential [36,37]. This includes optimising restocking frequencies, managing vial expiries, and reallocating stocks based on real-time demand. Our recommendations for adapting PEP management strategies using minimum restocking volumes and alert thresholds provide actionable guidelines for policymakers and should improve PEP availability and minimise stockouts, responding to anticipated shifts in healthcare-seeking behaviour and PEP demand spurred by Gavi’s investment.

In conclusion, we recommend adopting the ID 1-week regimen in all settings (no matter how few patients present to a clinic). We further suggest optimising stock management based on average patient throughput at facilities that supply PEP, and developing a feasible model for decentralizing PEP should address immediate access challenges for these emergency vaccines. Moreover, improved PEP monitoring and restocking should support countries to improve their preparedness and response capabilities for rabies and other public health emergencies.

## Supplementary Material

Supplementary data to this article can be found online at https://doi.org/10.1016/j.vaccine.2025.127108.

SupplementaryFigure1

SupplementaryFigure4

SupplementaryTable

figure SupplementaryFigure2

figure SupplementaryFigure3

## Figures and Tables

**Table 1:** Minimum restocking volumes and alert thresholds and IM given quarterly restocking. Stocking volumes and alert thresholds are shown for the 1-week ID regimen and compared to the IM, indicated in parentheses.

Average bites per month	Minimum restocking volume for ID (IM) vaccination	Minimum alert threshold for ID (IM) vaccination	Average annual restocks for ID (IM) vaccination
<5	100 (200)	60 (170)	1 (1)
<10	125 (375)	80 (310)	2 (1)
<20	225 (700)	120 (610)	1 (1)
<30	300 (1000)	160 (870)	1 (2)
<50	450 (1700)	240 (1450)	1 (1)
<75	675 (2650)	360 (2330)	1 (1)
<100	875 (3450)	440 (3030)	1 (1)

## Data Availability

The data and code are available on GitHub https://github.com/marthaluka/supply_chain_management (DOI: 10.5281/zenodo.15172122)
