# Supplementary figures and images for: Optimising human rabies vaccine supply chains: A modelling study
Source: Vaccine. Author manuscript; Available in PMC 2026 Apr 24. (PMC7619032; doi:10.1016/j.vaccine.2025.127108)

Year  
2018 2020 2022  
2019 2021 2023

Monthly Bites

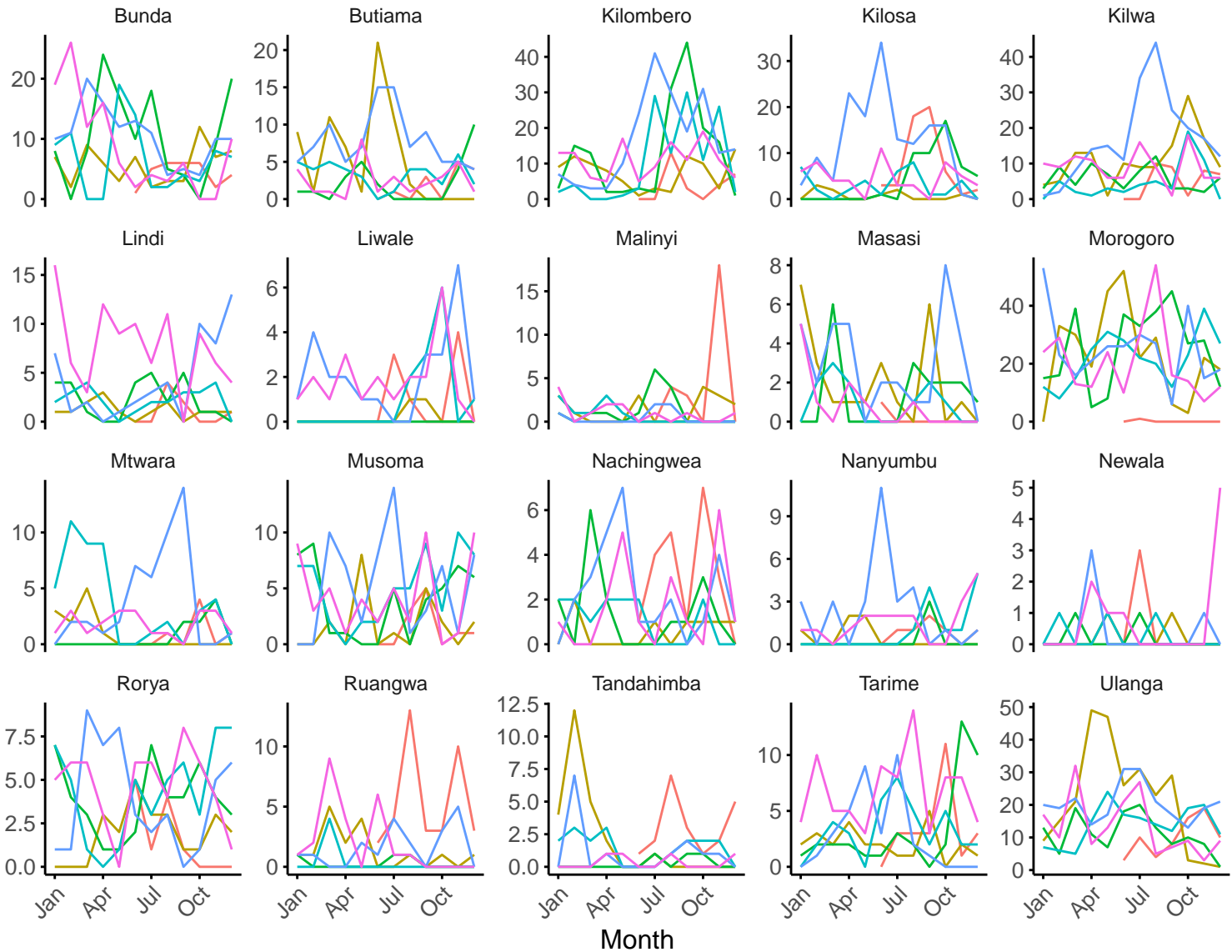

Supplement: SupplementaryFigure1 [file EMS213417-supplement-SupplementaryFigure1.pdf]

A

ID IM

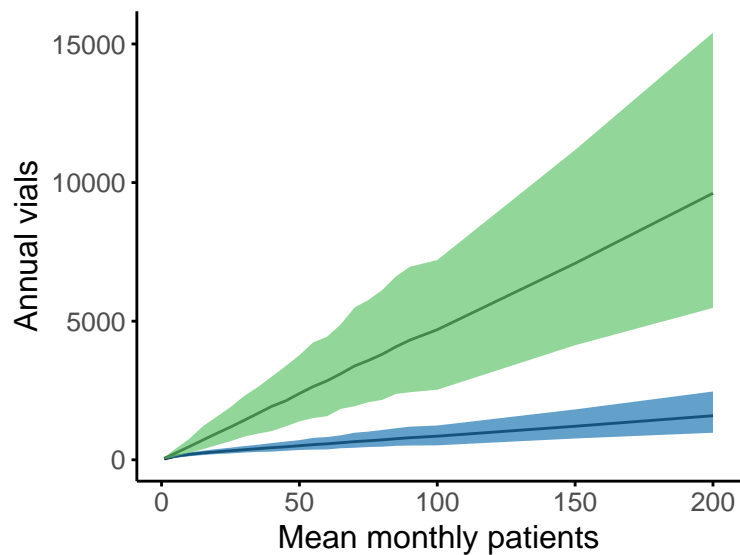

B

High Moderate None

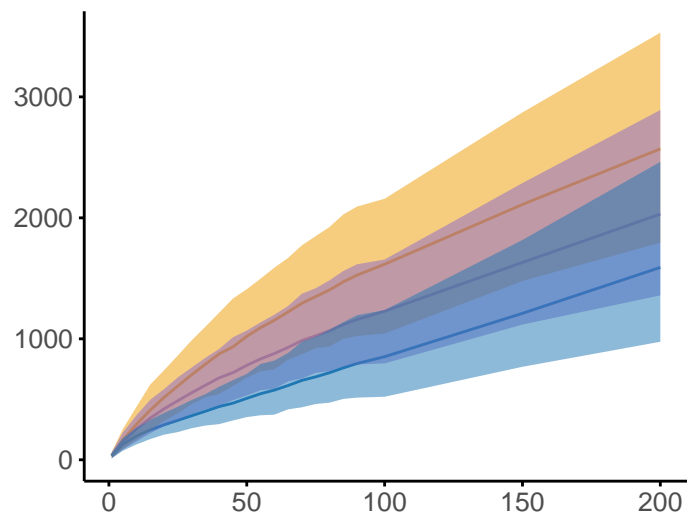

C

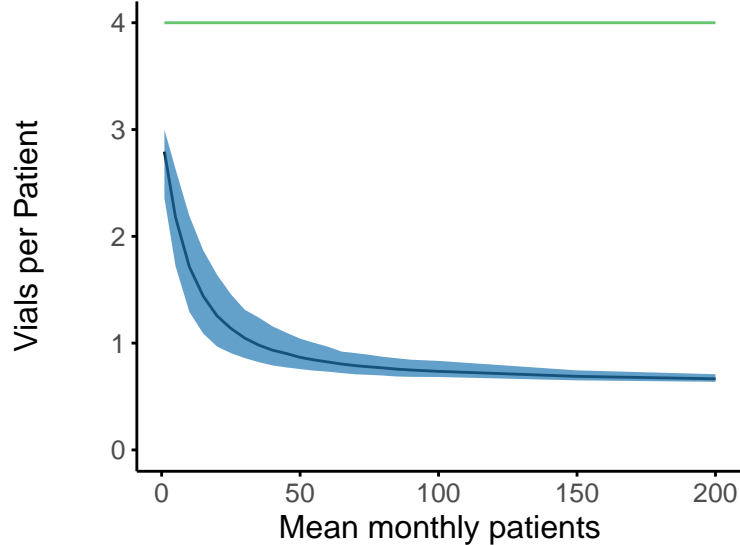

D

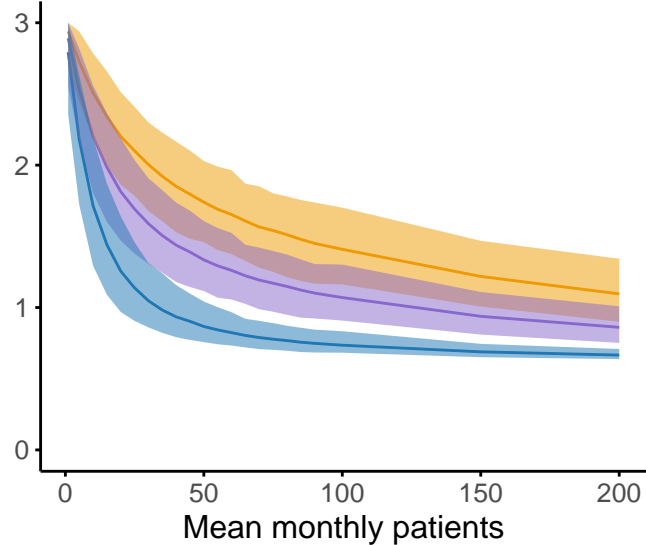

Supplement: SupplementaryFigure4 [file EMS213417-supplement-SupplementaryFigure4.pdf]

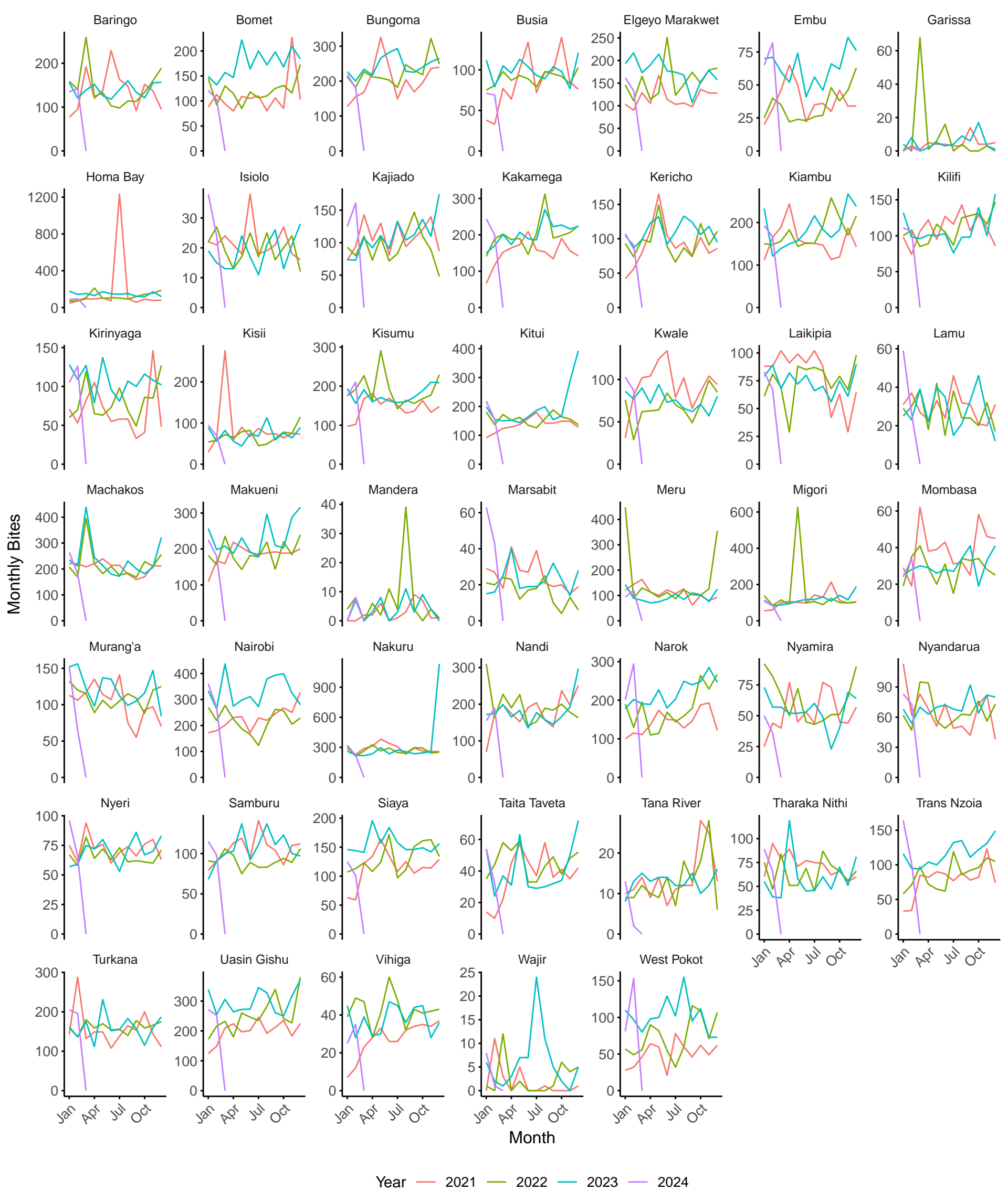

Supplement: figure SupplementaryFigure2 [file EMS213417-supplement-figure_SupplementaryFigure2.pdf]
