# Supplementary material for: Optimising human rabies vaccine supply chains: A modelling study
Source: Vaccine. Author manuscript; Available in PMC 2026 Apr 24. (PMC7619032; doi:10.1016/j.vaccine.2025.127108)
Supplement: SupplementaryTable [file EMS213417-supplement-SupplementaryTable.docx]

**Supplementary Figure 1.** Monthly bite patient dynamics across IBCM districts in Tanzania, coloured by year.

**Supplementary Figure 2.** Monthly bite patient dynamics across counties in Kenya, coloured by year.

**Supplementary Figure 3. Bite patient dynamics across Kenya.** (A) Map of Kenyan counties, highlighting the bite patients per capita. (B) Time series of monthly bite patient presentations, with a low-throughput sub-county, overlaid on a high-throughput sub-county for comparison. The black dotted line represents the average national monthly bite patient count. (C) Distribution of monthly bite cases (points), with a fitted negative binomial distribution line (solid lines) for both a low-throughput and a high-throughput sub-county. (D) Relationship between mean monthly bite patients and surge factor; the inset shows the distribution of surge factors nationally, with the line indicating the trend between surge factor and mean monthly bite patients, and the shaded polygon representing the 95% confidence interval around this trend.

**Supplementary Figure 4. Comparison of annual vial requirements and vials per patient across different patient throughput levels, PEP regimens, and decentralization scenarios**. A) Annual vial requirements by mean monthly patient presentations for intramuscular (IM) and intradermal (ID) regimens under a centralised distribution model. B) Vials per patient by mean monthly patient presentations for the IM and ID regimens, assuming a centralised scenario. C) Annual vial requirements by mean monthly patient presentations for the ID regimen under various decentralization levels (None, Moderate, High). D) Vials per patient for the ID regimen by mean monthly patient presentations under different decentralization levels

**Supplementary Table 1. WHO-recommended regimens for post-exposure vaccination investigated.**

| **Route of administration** | **Schedule (Days)** | **Vaccine volume per dose** |
| --- | --- | --- |
| Intradermal (ID) | 0, 3, 7 | 0.2 ml (2x 0.1ml injections) |
| Intramuscular (IM) | 0, 3, 7, 14 | 1 ml |

**Supplementary Table 2. Summary statistics of IBCM data by district**

| **District** | **Mean (Range) Monthly Bites** | **Proportion of months with zero bites** | **Surge factor** | **Total bites** | **Total deaths** | **Negative binomial parameters: size (mu)** | **Population served** | **Total patients** | **Number of facilities (range if varied over study period)** | **Proportion attending main PEP centre (range if varied over study period)** |
| --- | --- | --- | --- | --- | --- | --- | --- | --- | --- | --- |
| Bunda | 7.93 (0-26) | 0.09 | 2.7 | 531 | 5 | 1.77 (7.93) | 426,792 | 531 | 4 | 0.47 |
| Butiama | 3.97 (0-21) | 0.21 | 3.78 | 266 | 1 | 1.05 (3.97) | 281,656 | 266 | 3 (2-3) | 0.62 (0.53-0.83) |
| Kilombero | 10.73 (0-44) | 0.08 | 3.22 | 719 | 3 | 1.13 (10.73) | 582,960 | 719 | 4 (2-4) | 0.5 (0.41-0.52) |
| Kilosa | 5.25 (0-34) | 0.27 | 4.01 | 352 | 4 | 0.6 (5.26) | 617,032 | 352 | 4 (1-4) | 0.74 (0.68-1) |
| Kilwa | 9.06 (0-44) | 0.06 | 3.39 | 607 | 6 | 1.49 (9.06) | 297,676 | 607 | 2 | 0.87 (0.84-0.96) |
| Lindi | 3.24 (0-16) | 0.21 | 3.81 | 217 | 4 | 1.08 (3.24) | 340,619 | 217 | 3 (2-3) | 0.4 (0.36-0.76) |
| Liwale | 1.03 (0-7) | 0.56 | 5.83 | 69 | 0 | 0.61 (1.03) | 136,505 | 69 | 1 | 1 |
| Malinyi | 1.21 (0-18) | 0.56 | 3.88 | 81 | 7 | 0.44 (1.21) | 225,126 | 81 | 1 | 1 |
| Masasi | 1.45 (0-8) | 0.45 | 4.38 | 97 | 6 | 0.77 (1.45) | 452,363 | 97 | 2 (1-2) | 0.94 (0.92-1) |
| Morogoro | 20.76 (0-54) | 0.11 | 2.52 | 1,391 | 16 | 1.35 (20.76) | 859,145 | 1391 | 7 (5-7) | 0.54 (0.5-0.72) |
| Mtwara | 2.03 (0-14) | 0.44 | 5.1 | 136 | 6 | 0.55 (2.03) | 437,900 | 136 | 2 | 0.88 (0.87-0.94) |
| Musoma | 3.72 (0-14) | 0.24 | 2.69 | 249 | 0 | 1.05 (3.72) | 430,837 | 249 | 3 (2-3) | 0.52 (0.51-0.6) |
| Nachingwea | 1.55 (0-7) | 0.35 | 4.1 | 104 | 3 | 1.38 (1.55) | 233,655 | 104 | 2 (1-2) | 0.9 (0.87-1) |
| Nanyumbu | 1.1 (0-11) | 0.55 | 4.55 | 74 | 2 | 0.6 (1.1) | 204,323 | 74 | 1 | 1 |
| Newala | 0.34 (0-5) | 0.8 | 8.82 | 23 | 3 | 0.31 (0.34) | 241,288 | 23 | 1 | 1 |
| Rorya | 3.48 (0-9) | 0.14 | 2.3 | 233 | 1 | 2.96 (3.48) | 354,490 | 233 | 4 (2-4) | 0.57 (0.5-0.97) |
| Ruangwa | 1.55 (0-13) | 0.5 | 6.03 | 104 | 5 | 0.49 (1.55) | 185,573 | 104 | 1 | 1 |
| Tandahimba | 1.22 (0-12) | 0.52 | 5.74 | 82 | 1 | 0.58 (1.22) | 299,073 | 82 | 1 | 1 |
| Tarime | 3.61 (0-14) | 0.14 | 3.24 | 242 | 1 | 1.75 (3.61) | 537,891 | 242 | 5 (2-5) | 0.59 (0.43-0.63) |
| Ulanga | 15.39 (1-49) | 0 | 2.42 | 1,031 | 4 | 2.83 (15.39) | 232,895 | 1031 | 4 (3-4) | 0.57 (0.4-0.94) |
