# Supplementary material for: Optimising human rabies vaccine supply chains: A modelling study
Source: Vaccine. Author manuscript; Available in PMC 2026 Apr 24. (PMC7619032; doi:10.1016/j.vaccine.2025.127108)
Supplement: figure SupplementaryFigure3 [file EMS213417-supplement-figure_SupplementaryFigure3.pdf]

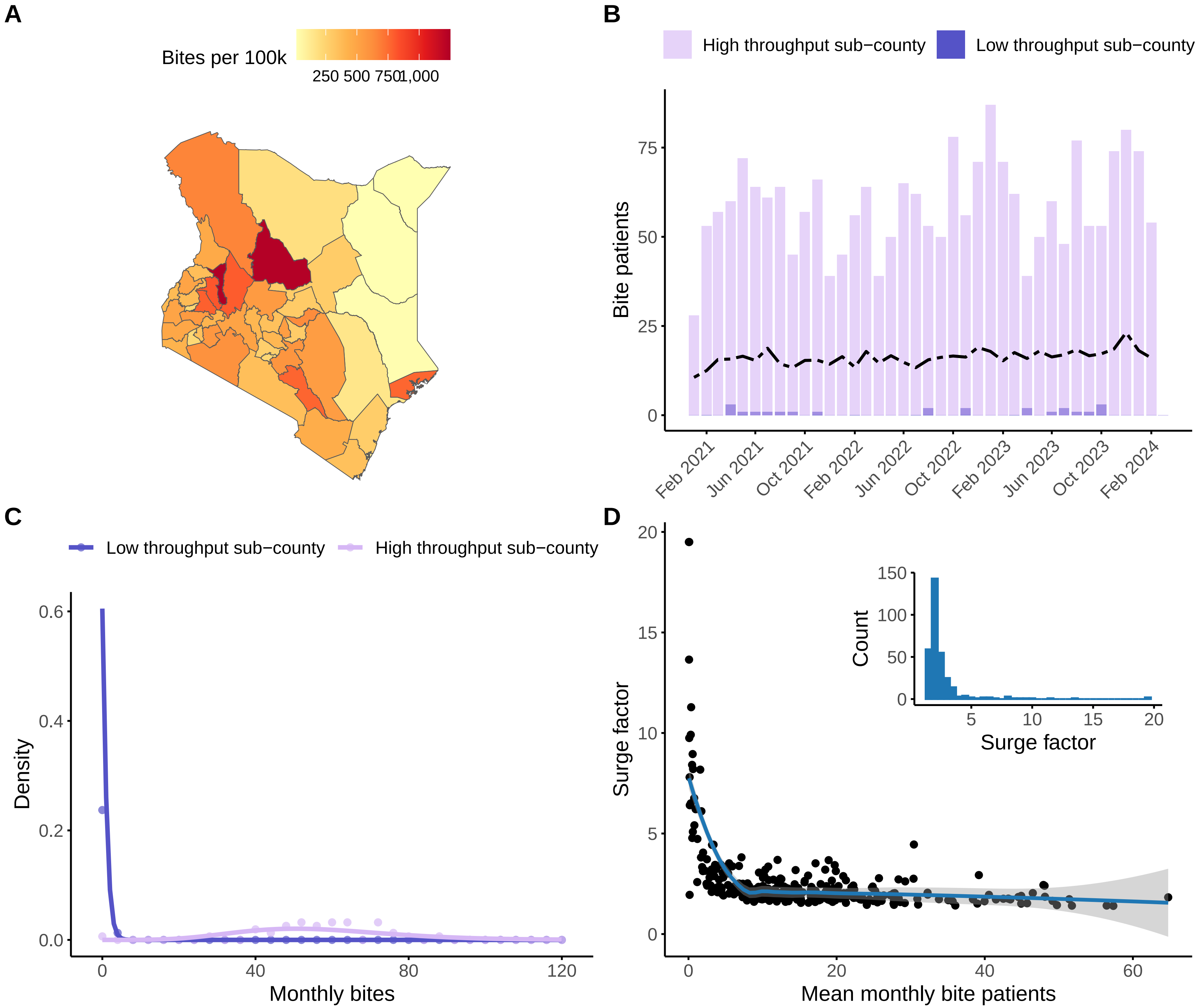
A
Bites per 100k
250 500 750 1,000
B
High throughput sub−county
Low throughput sub−county
Bite patients
0
25
50
75
Feb 2021
Jun 2021
Oct 2021
Feb 2022
Jun 2022
Oct 2022
Feb 2023
Jun 2023
Oct 2023
Feb 2024
C
Low throughput sub−county
High throughput sub−county
Density
0.0
0.2
0.4
0.6
0
40
80
120
Monthly bites
D
Surge factor
0
5
10
15
20
0
20
40
60
Mean monthly bite patients
Count
0
50
100
150
5
10
15
20
Surge factor
